# Live-cell protein labelling with nanometre precision by cell squeezing

**DOI:** 10.1038/ncomms10372

**Published:** 2016-01-29

**Authors:** Alina Kollmannsperger, Armon Sharei, Anika Raulf, Mike Heilemann, Robert Langer, Klavs F. Jensen, Ralph Wieneke, Robert Tampé

**Affiliations:** 1 Institute of Biochemistry, Biocenter, Goethe-University Frankfurt, Max-von-Laue Strasse 9, 60438 Frankfurt/Main, Germany; 2 Department of Chemical Engineering, David H. Koch Institute for Integrative Cancer Research, Massachusetts Institute of Technology (MIT), 500 Main Street, Building 76-661, Cambridge, Massachusetts 02139, USA; 3 Institute of Physical and Theoretical Chemistry, Goethe-University Frankfurt, Max-von-Laue Strasse 7, 60438 Frankfurt/Main, Germany; 4 Cluster of Excellence—Macromolecular Complexes, Goethe-University Frankfurt, Max-von-Laue Strasse 9, 60438 Frankfurt/Main, Germany

## Abstract

Live-cell labelling techniques to visualize proteins with minimal disturbance are important; however, the currently available methods are limited in their labelling efficiency, specificity and cell permeability. We describe high-throughput protein labelling facilitated by minimalistic probes delivered to mammalian cells by microfluidic cell squeezing. High-affinity and target-specific tracing of proteins in various subcellular compartments is demonstrated, culminating in photoinduced labelling within live cells. Both the fine-tuned delivery of subnanomolar concentrations and the minimal size of the probe allow for live-cell super-resolution imaging with very low background and nanometre precision. This method is fast in probe delivery (∼1,000,000 cells per second), versatile across cell types and can be readily transferred to a multitude of proteins. Moreover, the technique succeeds in combination with well-established methods to gain multiplexed labelling and has demonstrated potential to precisely trace target proteins, in live mammalian cells, by super-resolution microscopy.

## Abstract (web summary)

The direct observation of intracellular processes through microscopy can provide key insight into biological pathways. Here the authors demonstrate the combination of microfluidic cell squeezing and fluorescent label delivery to native proteins for high throughput live cell super-resolution imaging.

## Graphical abstract

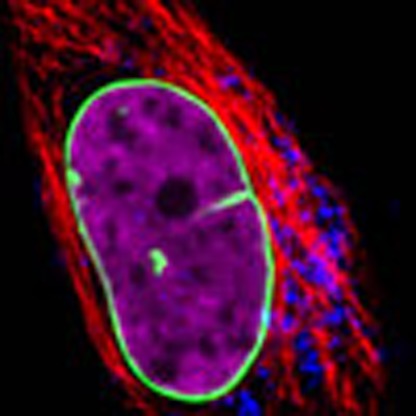

Direct observation of intracellular processes has the potential to yield insight into fundamental biological pathways and disease mechanisms. Several techniques have been developed to enable high-resolution imaging of live cells; yet, the limited ability to trace intracellular components has hindered progress. Hence, two of the persistent challenges are probe design and cellular delivery with minimal toxicity, pivotal for advances in live-cell imaging technologies. Here we describe an efficient approach to tag and image intracellular components in live mammalian cells. Using the microfluidic cell squeezing platform to deliver small fluorescent *tris*-*N*-nitrilotriacetic acid (*tris*NTA) probes (∼1 kDa), we demonstrate highly efficient, minimally disruptive, light-triggered tracing of native proteins and the subsequent super-resolution imaging of live-cell phenomena.

Live-cell microscopy contributed significant knowledge of dynamic processes such as protein trafficking and single-molecule localization-based imaging techniques visualize proteins with high-resolution information (≤50 nm)^1^,^2^. All fluorescent imaging techniques require protocols to introduce the label with the need to minimize its influence on the system. Fluorescent proteins, self-labelling tags^3^,^4^,^5^,^6^ or labelling by enzymatic methods^7^,^8^ can interfere with protein function, assembly or dynamics. Bulky fusion proteins (>20 kDa) entail the risk of steric hindrance and functional perturbations, whereas smaller tags (for example, tetracysteine tag) deal with unspecific interactions or require additional experimental steps^8^ and optimized flanking sequences for each protein target^9^. Although synthetic fluorophores have enhanced photostability, quantum yield, spectral range and localization precision, it is difficult to introduce such probes to the cytosolic environment using existing delivery technologies. On the one hand, current transduction strategies such as delivery by cell-penetrating peptides (CPPs), electroporation and so on are suboptimal, suffering from poor and endosomal uptake, rapid degradation by extracellular and endosomal proteases, low *in vivo* efficiency or elaborated chemical synthesis. On the other hand, antibody-based labelling approaches, for example, are limited to chemically arrested (fixed) cells and the availability of specific antibodies for a protein target. Owing to the described limitations of existing labelling and transduction technologies, there is a persistent demand for techniques enabling high-throughput in-cell labelling by minimal tags that are conductive to high-resolution and super-resolution microscopy.

Here we demonstrate robust in-cell targeting of native proteins using a labelled multivalent chelator head *tris*NTA^10^ and a genetically encoded oligohistidine sequence (Fig. 1a). *tris*NTA site specifically recognizes His_6–10_-tagged proteins in the (sub)nanomolar range (*K*_d_ of 0.1–10 nM) even in the crowded cellular environment^11^. The minimal size of the tag and the molecular probe allows direct targeting with nanometre precision at subnanomolar concentrations as required for single-molecule localization-based imaging techniques^1^,^2^,^12^,^13^ with no impact on intracellular trafficking or demand for additional cofactors affecting endogenous processes. We simplified efficient transfer of the *tris*NTA probe into living cells by cell squeezing^14^, combining precisely controlled cytosolic delivery with high specificity and low cytotoxicity. Briefly, transient cell permeabilization is achieved by rapid viscoelastic deformation of cells as they pass through micrometre-scale constrictions. This facilitates fast uptake of probes into the cytosol before cell-intrinsic repair mechanisms kick in^15^.

## Results

### High-affinity protein labelling at subnanomolar concentration

**Figure 1:**
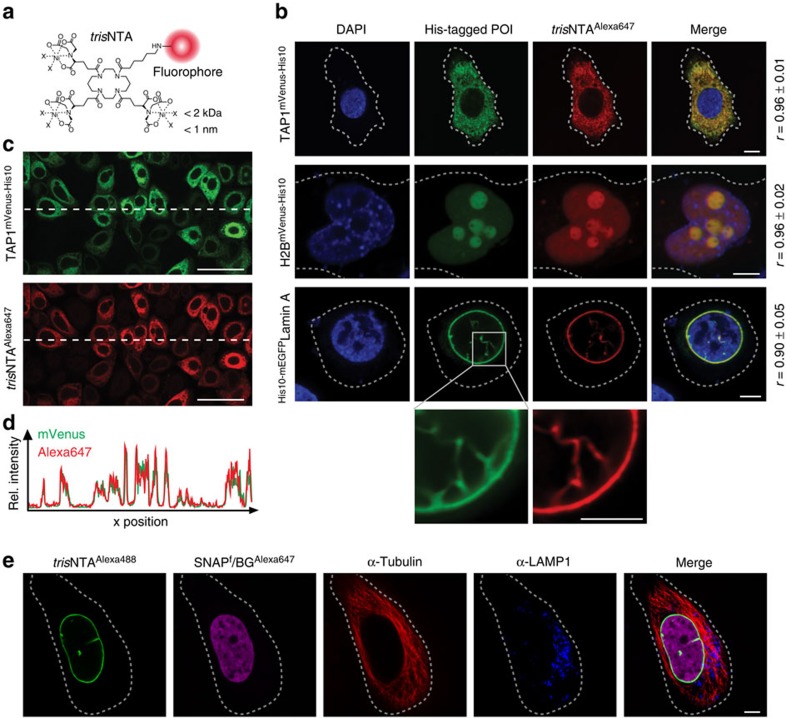
‘Traceless' tracing of protein assemblies by a minimal lock-and-key recognition pair. (**a**) Chemical structure of *tris*NTA conjugated to various organic fluorophores (red circle). (**b**) Subcellular tracing of His-tagged POIs by *tris*NTA^Alexa647^. Cells expressing TAP1^mVenus-His10^ (HeLa Kyoto), H2B^mVenus-His10^ (HeLa) or ^His10-mEGFP^Lamin A (Chinese hamster ovary (CHO-K1)) were fixed and stained with *tris*NTA^Alexa647^. Excellent co-localization (merge) between *tris*NTA^Alexa647^ (red) and all His-tagged POIs (green) was observed. Pearson's coefficients (*r*) were calculated from eight to ten individual images (right). Dashed lines indicate the cell border. (**c**) Specific labelling of TAP1^mVenus-His10^ by *tris*NTA^Alexa647^ in fixed HeLa Kyoto cells with a Pearson's coefficient of *r*=0.95. (**d**) Cross-section of relative fluorescence intensities (mVenus and Alexa647), indicated by a horizontal dashed line in **c** shows excellent correlation of His-tagged POI expression level and *tris*NTA^Alexa647^ staining. (**e**) Combination of the lock-and-key element with established labelling methods. HeLa Kyoto cells expressing ^His10^LaminA and H2B^SNAPf^ were simultaneously labelled with *tris*NTA^Alexa488^, benzyl guanine (BG)^Alexa647^ and antibodies against tubulin, as well as the lysosomal protein lysosomal-associated membrane protein 1 (LAMP1). Scale bars, 5 μm (**b**,**e**), 50 μm (**c**).

We first investigated the specificity of the *tris*NTA/His tag targeting in chemically arrested cells. To evaluate precise localization, different proteins resident at distinct subcellular compartments were selected: (i) the transporter associated with antigen processing (TAP) in the membrane of the endoplasmic reticulum^16^; (ii) histone 2B (H2B) in the nucleus; and (iii) Lamin A at the nuclear envelope. All proteins of interest (POIs) were fused to a His_10_ tag and a fluorescent protein (TAP1^mVenus-His10^, H2B^mVenus-His10^ and ^His10-mEGFP^Lamin A) for specific targeting and co-localization studies, respectively. For sensitive detection, *tris*NTA was covalently coupled to different fluorescent dyes (*tris*NTA^f^, f=Alexa488, ATTO565, ATTO647N, Alexa647 and ATTO655). Mammalian cells were transiently transfected with the corresponding target genes. His-tagged proteins were specifically stained by *tris*NTA^f^ with excellent co-localization and signal-to-noise ratio (Pearson's coefficients between 0.90 and 0.96), using confocal laser scanning microscopy (CLSM; Fig. 1b and Supplementary Fig. 1). Strikingly, even at 200 pM of *tris*NTA^f^, His-tagged proteins were labelled with high specificity (Supplementary Fig. 2). By analysing a variety of fluorescent dyes, we noticed that *tris*NTA^ATTO565^ targeting produced a higher background compared with *tris*NTA^ATTO647N^, *tris*NTA^Alexa647^ or *tris*NTA^ATTO655^ (Supplementary Fig. 3). This was assigned to unspecific binding of the ATTO565 dye. Moreover, the superposition of both fluorescence intensity profiles reflects an excellent correlation between the POI expression level and the labelling density of *tris*NTA^Alexa647^ (Fig. 1c,d, Pearson's coefficient *r*=0.95). Notably, using nanomolar concentrations, *tris*NTA^f^ labelling is significantly more efficient within 30 min than SNAP^f^-tag labelling (Supplementary Fig. 4). In contrast, mammalian cells expressing H2B lacking a His tag showed neither *tris*NTA^f^ labelling nor unspecific staining (Supplementary Fig. 5). In conclusion, *tris*NTA^f^ targeting at subnanomolar concentrations is highly specific to trace His-tagged proteins. To exploit these benefits further, we combined *tris*NTA^f^ with well-established labelling methods for multiplexed protein modification. Specific *tris*NTA^Alexa488^ labelling of ^His10^LaminA (Fig. 1e, green) was successfully achieved in combination with SNAP^f^-tag labelling of H2B (magenta) and antibody labelling of tubulin (red), as well as the lysosomal-associated membrane protein 1 (blue). Thus, the ultra-small interaction pair complements the toolbox of well-established labelling techniques and the nanomolar concentrations perform various avenues in multiplexed labelling.

### High-throughput live-cell labelling within mammalian cells

**Figure 2:**
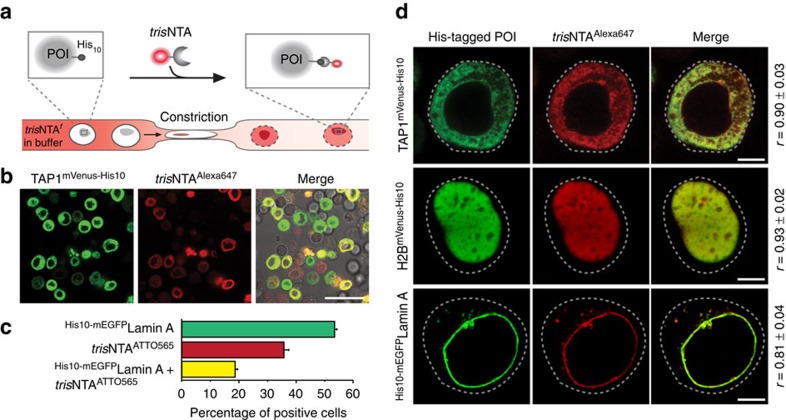
Live-cell labelling of protein assemblies in distinct subcellular compartments. (**a**) Delivery of *tris*NTA^f^ into living cells by microfluidic cell squeezing. Cells are pressed through micrometre constrictions of a microfluidic device, to introduce *tris*NTA^f^ (bottom). Cell passage causes the formation of transient holes in the plasma membrane and enables *tris*NTA^f^ transfer into the cytosol. His-tagged POI (grey) is specifically labelled by fluorophore-conjugated *tris*NTA (*tris*NTA^f^, top), resulting in co-localization at the respective subcellular compartment. (**b**) High-throughput in-cell labelling of TAP1^mVenus-His10^-transfected HeLa cells by *tris*NTA^Alexa647^ imaged 10 min after squeezing by CLSM. (**c**) Flow cytometry analysis of ^His10-mEGFP^Lamin A-transfected HeLa Kyoto cells, after transduction with 100 nM of *tris*NTA^ATTO565^. Fluorescence intensity profiles of mEGFP and ATTO565 revealed that 20% of all cells were double positive for the His-tagged POI and *tris*NTA^f^. The error bars indicate the s.d. of three experimental replicates. (**d**) Specific labelling of His-tagged proteins in living cells at diverse subcellular localizations. *tris*NTA^Alexa647^ was delivered to cells and transfected with TAP1^mVenus-His10^ (HeLa), ^His10-mEGFP^Lamin A (Chinese hamster ovary (CHO-K1)) or H2B^mVenus-His10^ (HEK293) by squeezing. High target specificity of *tris*NTA^Alexa647^ is represented by excellent co-localization with the POIs (merge). Determined Pearson's coefficients range from 0.81 to 0.93. Images were taken by CLSM 10 min (**b**) or 1 h (**d**) after squeezing. Dashed lines indicate the cell border. Scale bars, 50 μm (**b**), 5 μm (**d**).

Encouraged by these observations, we aimed at protein labelling in living cells. To transfer *tris*NTA^f^ into cells, we applied microfluidic cell squeezing (Fig. 2a). As the *tris*NTA^f^ probes are chemically diverse relating to the used fluorophores, common transduction strategies are unlikely to efficiently deliver nanomolar concentrations of *tris*NTA^f^ into mammalian cells. Specifically, mammalian cells were mechanically pushed (‘squeezed') through micrometre constrictions at elevated pressure of 30 psi. This approach allows for high cell survival (>90%) and efficient uptake of *tris*NTA^f^ (up to 80%; Supplementary Figs 6 and 7). Energy-dependent endocytosis, often observed at cargo transfer with supercharged molecules (Supplementary Fig. 8) or low concentrations of CPPs (Supplementary Fig. 9)^17^, were prevented by performing cell squeezing at 4 °C (Supplementary Fig. 10).

By squeezing TAP1^mVenus-His10^-transfected HeLa cells in the presence of *tris*NTA^f^ (100 nM), we achieved a high-throughput delivery and a high-density labelling, illustrated by an excellent co-localization between both reporter molecules (Fig. 2b). We noticed that both probe delivery by cell squeezing and protein labelling are highly reproducible (*n*>20). To quantify the *tris*NTA^f^ delivery, we performed flow cytometry analysis on micromanipulated cells. Thirty-five per cent of transfected cells (54% of total) were effectively transduced with *tris*NTA^ATTO565^ (Fig. 2c), which is > 30 × more efficient for *tris*NTA^f^ delivery than electroporation (Supplementary Fig. 11). Squeezing of up to 1,000,000 cells per second enables live-cell labelling at high throughput and reproducibility, and hence largely exceeds the efficiency achieved by other direct transfer methods such as microinjection. The massively parallel and constant cell transduction surpasses the stochastic, while variable efficient uptake by CPPs (Supplementary Fig. 9) and is more than 1,000-fold below the micromolar probe concentrations used by elegant self-labelling tags, for example, SNAP tag^3^,^4^,^5^,^6^.

### Highly specific protein targeting with minimal disturbance

Analogous micromanipulation and live-cell labelling was applied to H2B^mVenus-His10^- and ^His10-mEGFP^Lamin A-transfected cells (Fig. 2d). Cell transduction was analysed 15 min and 1 h after squeezing by CLSM. An excellent co-localization between the *tris*NTA^f^ reporter molecule and the His-tagged POIs was observed (Pearson's coefficients range from 0.81 to 0.93), in line with the subcellular localization in chemically arrested cells. Notably, live-cell labelling is independent of cell types, for example, HeLa, HeLa Kyoto, Chinese hamster ovary (CHO-K1) or human embryonic kidney 293 cells (Figs 1b and 2d). Beyond that, *tris*NTA^f^ labelling after removal of the bulky fluorescent protein fully exploited the small size of the lock-and-key element and confirmed again specific labelling in living cells with minimal perturbation (Supplementary Fig. 12). The specificity was validated in cells transfected with H2B^EGFP^ lacking a His tag and untransfected cells (Supplementary Fig. 13). In both cases, neither co-localization of *tris*NTA^f^ with H2B^EGFP^ nor unspecific binding was detected. Delivery of nickel-free *tris*NTA^f^ or of free Alexa647 dye showed no labelling in TAP1^mVenus-His10^-transfected cells (Supplementary Figs 14 and 15). In contrast, *tris*NTA^Alexa647^ (Fig. 2 and Supplementary Fig. 14) and *tris*NTA^ATTO565^ (Supplementary Fig. 16) clearly stain TAP1^mVenus-His10^ at the endoplasmic reticulum membrane after cell squeezing. Noticeably, cell viability of *tris*NTA^f^-transduced cells was negligibly affected 1 and 24 h after labelling. Similar concentrations of unbound nickel ions inside mammalian cells had no significant toxic effects (Supplementary Fig. 7). In contrast, electroporation entailed more than twofold increased toxicity compared with squeezing (Supplementary Figs 7 and 11). Collectively, nanomolar delivery of *tris*NTA^f^ fully realized the potential of in-cell protein manipulation with minimal perturbation and modification rates exceeding common approaches.

**Figure 3:**
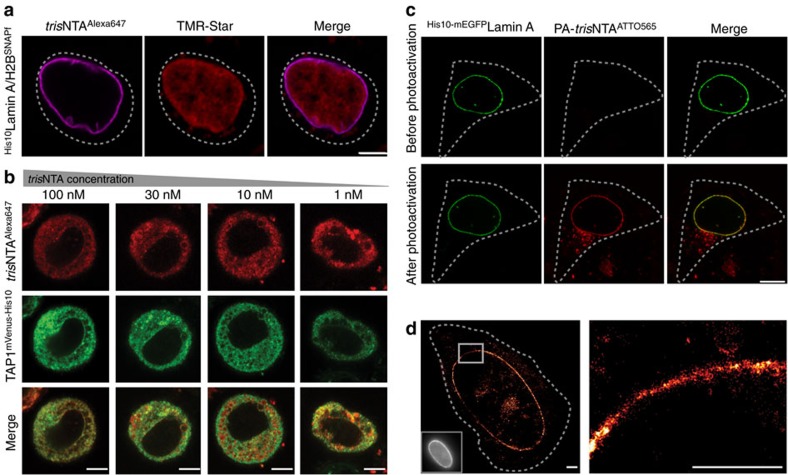
Light-triggered live-cell labelling and super-resolution microscopy of protein assemblies. (**a**) Combination of *tris*NTA labelling with SNAP^f^-tag labelling in living cells. HeLa Kyoto cells co-expressing ^His10^LaminA and H2B-SNAP^f^ were squeezed in the presence of 100 nM *tris*NTA^Alexa647^ (magenta) and subsequently incubated with cell-permeable TMR-Star (red) for SNAP^f^-tag labelling. Confocal images were taken 1 h after squeezing and demonstrate simultaneous specific labelling of the His-tagged LaminA and the SNAP^f^-tagged H2B. (**b**) *tris*NTA^Alexa64*7*^ concentration scan for tunable labelling of TAP1^mVenus-His10^ in HeLa Kyoto cells. High labelling density was obtained even at 1 nM *tris*NTA^Alexa647^ during squeezing. (**c**) Light-activated labelling of ^His10-mEGFP^Lamin A with PA-*tris*NTA^ATTO565^ in living HeLa Kyoto cells. Before photoactivation, no decoration of Lamin A was observed 24 h after squeezing (top), whereas on illumination fluorescence increase and specific labelling were monitored (bottom). (**d**) Reconstructed *d*STORM image of ^His10-mEGFP^Lamin A labelled with *tris*NTA^ATTO655^ (100 nM) in a living HeLa Kyoto cell. Increased spatial resolution (≤40 nm) was obtained in live-cell super-resolution imaging of *tris*NTA^ATTO655^ by *d*STORM (left and magnification right) compared with the wide-field image (left corner, bottom). Images were taken by CLSM (**a**–**c**) or *d*STORM (**d**) 1 h (**a**,**b**,**d**) or 24 h (**c**) after squeezing. Dashed lines indicate the cell border. Scale bars, 5 μm (**a**,**b**), 10 μm (**c**) and 2 μm (**d**).

After successful in-cell labelling of different His-tagged proteins, we aimed for *in vivo* multiplexed labelling by combining *tris*NTA^f^ with well-established labelling methods. By *tris*NTA^Alexa647^ delivery via squeezing and subsequent SNAP^f^-tag labelling, we achieved specific and distinct targeting of ^His10^LaminA in the presence of two different SNAP^f^-tagged proteins in live cells (Fig. 3a and Supplementary Fig. 17). Hence, *tris*NTA^f^ complements the toolbox for *in vivo* multiplexed labelling, offering minimal disturbance due to its small size and simultaneously using low nanomolar concentrations.

We next determined the minimal reporter concentration required for specific live-cell labelling. Well-resolved images of TAP1^mVenus-His10^ were obtained even at 1 nM of *tris*NTA^Alexa647^ (Fig. 3b). Based on previous observations, approximately one-third of the cargo provided during squeezing is the effective intracellular concentration^14^. Thus, the estimated cytosolic concentration of *tris*NTA^f^ further corroborates the high target sensitivity at subnanomolar concentrations (∼300 pM). These results are in line with the detection limit of ∼200 pM *tris*NTA^Alexa647^ in chemically arrested cells (Supplementary Fig. 2). Hence, this enables the precise adjustment of the effective, intracellular *tris*NTA^f^ concentration to improve the signal-to-background ratio, hardly realized by alternative approaches at nanomolar probe concentrations (for example, CPPs, SNAP, CLIP and Halo tag; Supplementary Figs 4 and 9)^3^,^4^,^6^,^18^, and circumvents endocytic uptake observed with supercharged proteins at similar nanomolar concentrations (Supplementary Fig. 8)^19^.

### In-cell protein modification with nanometre precision

Incited by this observation, we aimed at temporal and spatial control of protein tracing by light, which depends on low probe concentrations for high signal-to-background ratios. Using photoactivatable *tris*NTA^f^ (PA-*tris*NTA^ATTO565^)^20^ for dynamic cellular imaging on demand, light-activated *in vivo* labelling of ^His10-mEGFP^Lamin A was demonstrated up to 24 h after squeezing (Fig. 3c). Notably, already a 10-s 405-nm light pulse sufficiently activated PA-*tris*NTA at single-cell level and led to excellent co-localization in a dose-adapted manner. This probe enables *in situ* labelling at defined time points such as certain mitotic phases and paves the way for live-cell protein tracing with high temporal resolution. The nanomolar concentrations (≤10 nM) and in particular the small size of the tag and probe are especially beneficial for advanced microscopy techniques, bringing the fluorophore in 1-nm proximity to the target protein. Hence, we performed live-cell super-resolution microscopy with *tris*NTA^ATTO655^ on ^His10-mEGFP^Lamin A-transfected cells. Using direct stochastic optical reconstruction microscopy (*d*STORM)^21^, Lamin A structures with a high signal-to-noise ratio were obtained in the super-resolved images of live cells. A localization precision of 16.4±3.1 nm was achieved, resulting in a resolution of 40 nm by in-cell *tris*NTA^ATTO655^ tracing with substantially increased resolution compared with diffraction-limited fluorescence microscopy (Fig. 3d).

## Discussion

We established a high-throughput method for protein labelling inside living cells using a minimalistic lock-and-key probe. Our method is versatile in the choice of the molecular probe, cell type and the subcellular localization of the POIs, a persistent challenge in live-cell analysis. The high-affinity *tris*NTA/His tag interaction pair enables fast labelling (≤10 min) at subnanomolar concentrations with tunable labelling density and flexibility of cell-impermeable organic fluorophores. Compared with carrier-mediated transport by CPPs^11^,^18^, delivery of 1,000-fold lower concentrations (nM versus μM) effectively decreases the fluorescence background. Furthermore, high-throughput analysis with up to 1,000,000 cells per second can be achieved in contrast to microinjection. In addition, *tris*NTA^f^ delivery via squeezing avoids endocytic cargo uptake, frequently observed with low CPP concentrations and supercharged molecules, offering decreased toxicity and a >30-fold higher efficiency compared with electroporation. The minimal probe complements the toolbox of well-established labelling techniques such as self-labelling enzymes and can be combined with the latter to achieve distinct labelling of different proteins in fixed as well as in living cells. Moreover, *in situ* photoactivation of PA-*tris*NTA allows labelling at defined time points, to trace proteins for dynamic cellular imaging. The achieved close target proximity of the labelling pair substantially improved the localization accuracy in live-cell super-resolution microscopy. Remarkable aspects of our approach are the speed, flexibility and efficiency for high-throughput live-cell targeting of proteins even if assembled in stable and transient macromolecular complexes. This study is exploited via one of the smallest high-affinity lock-and-key recognition pairs known so far and allows even multiple cargos to be delivered simultaneously, displaying diverse chemical properties. The quantity of cargo for in-cell manipulation can be precisely tuned and the biological output can in turn be fine-tuned. As the affinity tag is widely used in life sciences and our delivery platform is broadly applicable across cell types, this live-cell labelling method could potentially be implemented across numerous cell-impermeable probes and prodrugs, as well as translated to difficult cell lines including patient-derived cells and embryonic stem cells, providing the opportunity to use these cells for advanced microscopy techniques and live-cell analysis.

## Methods

### Plasmid construction

The H2B construct H2B^mVenus-His10^ was generated by consecutive insertion of H2B and mVenus-His_10_ into pCDNA3.1(+) (Life Technologies). mVenus-His_10_ was PCR amplified using Phusion High-Fidelity DNA Polymerase (Fermentas) and the primer pair forward (fw) 5′-GCGCGCGCGGCCGCGTGAGCAAGGGCGAGGAGCTGTTCA-3′ (NotI restriction site underlined) and reverse (rev) 5′-GCGCGCTCTAGATTAGTGATGGTGGTGATGATGATG-3′ (XbaI restriction site underlined), and cloned into the pCDNA3.1(+) plasmid using the indicated restriction enzymes (Fermentas). Subsequently, H2B was amplified using the primer pair fw 5′-GCGCGCGGTACC**ATG**CCAGAGCCAGCGAAGTCTGCTCCCGC-3′ (Acc65I restriction site underlined, start codon bold) and rev 5′-GCGCGCGCGGCCGCTCTTGG AGCTGGTGTACTTAGTGAC-3′ (NotI restriction site underlined), and cloned into the pCDNA3.1(+) plasmid, amino terminally of mVenus-His_10_. As a template for the amplification of H2B, the plasmid pEGFP-N1 containing the human H2B sequence (plasmid 11680, Addgene) was used, which also served as control for the specificity of *tris*NTA^f^ for His-tagged POIs in living cells (Supplementary Figs 5 and 13). The plasmid encoding for human Lamin A was generously provided by Dr Sascha Neumann (Institute of Biochemistry, University of Cologne)^22^ and used as template to amplify Lamin A with the primer pair fw 5′-GCGCGCCTCGAGCTATGGAGACCCCGTCCCAGCGGCGCGCCACCCG-3′ (XhoI restriction site underlined) and rev 5′-GCGCGCGATATC**TTA**CATGATGCTGCAGTTCTGGGGGCTCTGGG-3′ (EcoRV restriction site underlined, stop codon bold). Lamin A was inserted into the pcDXC3GMS plasmid (Addgene) already containing His_10_-mEGFP, leading to the fusion gene encoding for ^His10–mEGFP^Lamin A. To generate the plasmid containing ^His10^Lamin A without a fluorescent protein, Lamin A was amplified via PCR, simultaneously introducing a His_10_-tag with the primer pair fw 5′-GCGCGCGGATCCACC**ATG***CACCATCATCATCATCATCACCACCATCAC*TCCGGACTCAGATCTCGAGTCATGGAGACCC-3′ (BamHI restriction site underlined, start codon bold, His_10_-tag italic) and rev 5′-GCGCGCGCGGCCGC**TTA**CATGATGCTGCAGTTCTGGGGGCTCTGGG-3′ (NotI restriction site underlined, stop codon bold). Indicated restriction sites were used to insert this PCR product into the pCDNA3.1 (+) vector. The pSNAP^f^-H2B and pSNAP^f^-Cox8A plasmids (New England Biolabs) were used for SNAP^f^-tag labelling. In addition, a plasmid coding for the core domain of TAP1, tagged with mVenus-His_10_ (TAP1^mVenus-His10^) was used as previously described^23^.

### Cell culture and transfection

HeLa cells, HeLa Kyoto cells, Chinese hamster ovary (CHO-K1) cells and human embryonic kidney 293 cells were maintained in DMEM medium with 4.5 g l^−1^ glucose (Gibco), supplied with 10% (v/v) FCS (Gibco) in T75 cell culture flasks (Greiner). Every 2–3 days, cells were passaged using PBS (Sigma-Aldrich) and 0.5% trypsin/0.02% EDTA/PBS (GE Healthcare). All cell lines were cultivated in a humidified tissue culture incubator at 37 °C and 5% CO_2_. Mycoplasma contamination tests were carried out regularly, following the guidelines described^24^. Transient transfection was performed with Lipofectamine 2000 (Life Technologies), following the manufacturer's instructions. For fixation and staining, 2 × 10^4^ cells per well were seeded into eight wells on cover glass II slides (Sarstedt) and transfected with 0.2 μg DNA per well. For squeezing experiments, 8 × 10^5^ cells were seeded into six-well cell culture plates (Greiner) and transfected with 2 μg DNA per well. After transfection, cells were incubated 12–48 h at 37 °C and 5% CO_2_ until experiments were performed.

### Cell viability test

To analyse cell viability after squeezing, the Sytox Blue Dead Cell stain (Life Technologies) was used to stain cells with a permeable plasma membrane. Cells were squeezed in the presence of 100 nM *tris*NTA^Alexa647^, 100 nM Alexa647 dye or 500 nM NiCl_2_, followed by incubation with 1 μM of Sytox Blue Dead Cell Stain (20 min, room temperature (RT)) at different time points. Cell viability analysis was performed using the Attune flow cytometer (Life Technologies) and data were processed using FlowJo 7.6.5 (Tree Star Inc.). Before detaching the cells, the supernatant was collected to avoid altering the results by removing dead cells during washing. Identical Sytox Blue Dead Cell Staining was conducted with cells after electroporation (described above), followed by flow cytometry analysis of cell viability and uptake of *tris*NTA^ATTO565^. All experiments were performed in triplicates and error bars indicate the s.d.

### Confocal imaging

Imaging was performed using the confocal laser scanning TCS SP5 microscope (Leica) and a Plan-Appochromat 63 × 1.4 Oil differential interference contrast objective. Images were acquired sequentially to avoid cross-talk. The following laser lines were used for excitation: 405 nm (diode laser) for 4,6-diamidino-2-phenylindole, Hoechst and Sytox Blue Dead Cell Stain; 488 nm (argon laser) for monomeric enhanced green fluorescent protein (mEGFP), Alexa488, MitoTracker^Green^ and Syto16; 488 or 514 nm (argon laser) for mVenus; 565 nm (diode-pumped solid-state laser) for ATTO565 and TMR-Star; and 633 nm (helium-neon laser) for ATTO647N, Alexa647 and ATTO655. For image analysis, ImageJ^25^ was used in combination with Fiji^26^.

### Super-resolution imaging

A custom-built microscope was used for super-resolution imaging of *tris*NTA^ATTO655^-labelled ^His10-mEGFP^Lamin A^27^. Samples were illuminated with 488 nm (Sapphire 488 LP, Coherent) and 643 nm (iBeam smart, Toptica Photonics) laser beams in total internal reflection fluorescence mode. The excitation light was focused on the back focal plane of a 100 × oil objective (PLAPO 100 × , total internal reflection fluorescence mode, numerical aperture 1.45, Olympus) mounted on an inverted microscope (Olympus IX71). The emission was recorded using an electron multiplying charge-coupled device camera (Ixon3, Andor) with frame-transfer mode, 5.1 × pre-amplifier gain and electron multiplying (EM) gain set to 200. For every sample, 40,000 images were recorded at a frame rate of 33 Hz and image reconstruction was performed with rapidSTORM^28^. The localization (*σ*_loc_) precision of the *d*STORM images was calculated to 16.4±3.1 nm and a resolution of ≤40 nm (for *d*STORM image see Fig. 3d). Calculations were performed according to Mortensen *et al.*^29^

### Fixation and *tris*NTA^f^ labelling

Before fixation, cells were washed with PBS (Sigma-Aldrich). Fixation with 4% formaldehyde (Roth)/PBS for 15 min at RT was followed by quenching using 50 mM glycine/PBS (10 min, RT; Roth) and permeabilization with 0.1% Triton X-100/PBS (10 min, RT; Roth). After blocking with 5% (w/v) BSA (Albumin Fraction V, Roth) in PBS (1 h, RT), cells were stained with 100 nM of *tris*NTA^f^ in 1% (w/v) BSA/PBS. Cells were stained with 0.1 μg ml^−1^ 4′,6-diamidino-2-phenylindole (Sigma-Aldrich) in 1% BSA/PBS for 30 min–1 h at RT. After washing with 5% BSA/PBS (3 × ), cells were postfixed with 2% formaldehyde/PBS (15 min, RT) and stored in PBS until confocal imaging was performed. When *tris*NTA^f^ labelling was combined with antibody labelling, 100 nM *tris*NTA was applied together with the primary antibodies for 1 h at RT. After subsequent washing with PBS, cells were incubated with secondary antibodies. Antibodies were all diluted in 1% BSA/PBS as follows: rabbit α-tubulin (Abcam, 1:350), mouse α-human CD107a (LAMP-1; Biolegend, 1:1,000), goat α-rabbit^Cy3^ (Abcam, 1:1,000) and goat α-mouse^PacificBlue^ (Life Technologies, 1:1,000). *tris*NTA was conjugated with the corresponding fluorescent dyes by reacting amino-*tris*NTA with the N-hydroxysuccinimide (NHS)-activated dye, respectively. After RP-C_18_ HPLC chromatography, the multivalent chelator head was loaded with Ni^2+^ (10 mM NiCl_2_ in 20 mM HEPES, pH 7.0) and purified by anion exchange chromatography (HiTrap Q HP; GE Healthcare)^11^.

### SNAP^f^-tag labelling in fixed cells

HeLa Kyoto cells transfected with H2B^SNAPf^ were labelled with O6-benzyl guanine^Alexa647^ (New England Biolabs) 24 h after transfection. After washing with PBS, cells were fixed with 4% formaldehyde/PBS (10 min, RT), washed twice with PBS and subsequently permeabilized using 0.1% Triton X-100/PBS (10 min, RT). Cells were washed three times with 0.5% BSA/PBS and incubated with 10 or 100 nM of benzyl guanine^Alexa647^ for 30 min at 37 °C. After three washing steps with 0.1% Triton X-100/0.5% BSA/PBS and two washing steps with PBS, imaging was conducted by CLSM. In case of combined *tris*NTA^f^- and SNAP^f^-tag labelling, SNAP^f^-tag labelling was performed first, followed by *tris*NTA^f^ labelling as described above.

### *tris*NTA^f^ delivery via cell squeezing

Squeezing was performed using a chip with constrictions of 7 μm in diameter and 10 μm in length (CellSqueeze 10-(7)x1, SQZbiotech), if not otherwise stated. In all microfluidic experiments, a cell density of 1.5 × 10^6^ cells per ml in 10% (v/v) FCS/PBS were squeezed through the chip at a pressure of 30 psi. Transduction was conducted at 4 °C, to block cargo uptake by endocytosis. During squeezing, the following cargo concentrations were used: 0.1–100 nM of *tris*NTA^f^ (f=ATTO565, ATTO647N, Alexa647 or ATTO655), 200 nM of PA-*tris*NTA^ATTO565^ and 100 nM of free Alexa647 (Molecular Probes). After squeezing, cells were incubated for 5 min at 4 °C, to reseal the plasma membrane^15^. Squeezed cells were washed with DMEM containing 10% FCS and 10 mM histidine (Sigma-Aldrich), to remove unspecifically bound *tris*NTA^f^ from the cell surface, seeded into eight wells on cover glass II slides (Sarstedt) in DMEM containing 10% FCS and cultured at 37 °C and 5% CO_2_. Confocal imaging was performed at different time points (0.25, 0.5, 1, 2 and 24 h) after squeezing. As a control for endosomal uptake, cells were incubated with 100 nM of *tris*NTA^f^ at RT and 4 °C without microfluidic cell manipulation (Supplementary Fig. 10). In case of combined *tris*NTA^f^ and SNAP^f^-tag labelling, cells were first squeezed in the presence of 100 nM *tris*NTA^Alexa647^ and 3 μM SNAP-Cell TMR-Star (New England Biolabs) was added 5 min after squeezing. After 15 min incubation at 37 °C and 5% CO_2_, cells were washed with 10% FCS/DMEM and incubated again 30 min under standard cell culture conditions before CLSM imaging was performed. To visualize mitochondria in case of Cox8A^SNAPf^ labelling MitoTracker^Alexa488^ was added 10 min prior imaging.

### *tris*NTA^f^ delivery by the CPP Tat_49–57_

TAP^mVenus-His10^-transfected HeLa Kyoto cells were incubated with different concentrations (10 μM and 100, 10 and 1 nM) of a non-covalent complex composed of Tat_49–57_^His6^ and *tris*NTA^Alexa647^ for 30 min. After washing with 10% FCS/DMEM and PBS at 37 °C, *tris*NTA uptake and labelling of His_10_-tagged TAP was analysed by live-cell imaging via CLSM^11^.

### *tris*NTA^f^ delivery via supercharged GFP (GFP^36+^)

*tris*NTA^ATTO565^ (100 nM) and ^His6^GFP^36+^ (100 nM) were incubated for 30 min at RT to form the *tris*NTA-His tag complex. HeLa cells were washed with PBS and treated with the pre-formed complex at 37 °C. *In vivo* uptake was immediately followed by CLSM. After 20 min, cells were washed three times with PBS and 20 U ml^−1^ heparin/PBS (2 × ), to remove the complex from the plasma membrane. Internalization of *tris*NTA/^His6^GFP^36+^ was analysed by CLSM after washing three times with PBS. ^His6^GFP^36+^ was expressed in BL21(DE3) *Escherichia coli.* After lysis by sonication in 2 M NaCl/PBS, ^His6^GFP^36+^ proteins were purified via immobilized metal ion affinity chromatography using Ni Sepharose 6 Fast Flow (GE Healthcare). Elusion was performed with 500 mM imidazole before desalting of the eluted protein was conducted with PD-10 desalting columns (GE Healthcare)^19^.

### *tris*NTA^f^ delivery via electroporation

Trypsinized HeLa Kyoto cells were permeabilized via electroporation in the presence of *tris*NTA^ATTO565^ (100 nM) with a Nucleofector Device (Lonza) using Nucleofector Kit V and program I-013. Transduced cells were washed with 10% FCS/DMEM and plated into eight-well cover glass II slides. To quantify *tris*NTA^f^ uptake, flow cytometry analysis (as described above) and CLSM imaging was performed 1 h after electroporation. Before CLSM imaging, transduced HeLa cells were incubated with Sytox Blue Dead Cell Stain or Syto16 (Life Technologies) live cell stain, to distinguish between dead and live cells. Cell viability was analysed by flow cytometry using Sytox Blue Dead Cell Stain (see above).

### Photoactivation of PA-*tris*NTA^f^

HeLa Kyoto cells, transfected with ^His10-mEGFP^ Lamin A, were squeezed in the presence of PA-*tris*NTA^ATTO565^ (200 nM)^20^ and incubated at 37 °C and 5% CO_2_. Imaging via CLSM and photoactivation was conducted 2 or 24 h after squeezing, after cells reattached to the glass surface. PA-*tris*NTA^ATTO565^ was photoactivated in single cells by illumination with the 405-nm laser for 10 s. Imaging of both channels (mEGFP and ATTO565) was performed before and directly after photoactivation.

## Additional information

**How to cite this article:** Kollmannsperger, A. *et al.* Live-cell protein labelling with nanometre precision by cell squeezing. *Nat. Commun.* 7:10372 doi: 10.1038/ncomms10372 (2016).

## Supplementary Material

Supplementary Information Supplementary Figures 1-17
