# Supplementary material for: Live-cell protein labelling with nanometre precision by cell squeezing
Source: Nat Commun. 2016 Jan 29;7:10372. doi: 10.1038/ncomms10372 (PMC4740111; doi:10.1038/ncomms10372)
Supplement: Supplementary Information — Supplementary Figures 1-17 [file ncomms10372-s1.pdf]

Supplementary Information

SUPPLEMENTARY FIGURES

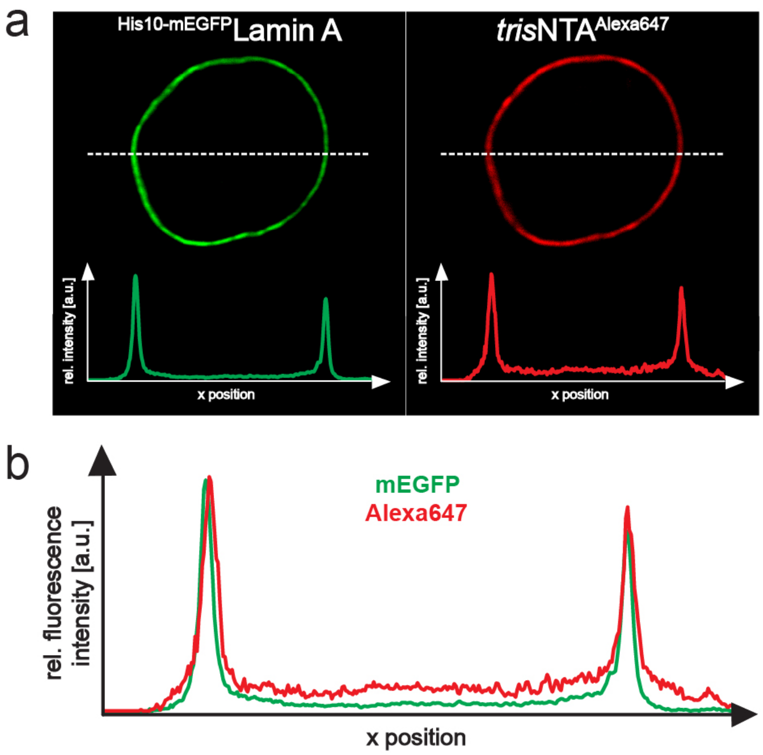

Supplementary Figure 1 ı Cross-section of relative fluorescence intensities. (**a**) HeLa Kyoto cells transiently transfected with $^{\text{His10-mEGFP}}$Lamin A (green) were chemically arrested with 4% paraformaldehyde and stained with *tris*NTA$^{\text{Alexa647}}$ (100 nM, red). Horizontal dashed lines indicate the analyzed fluorescence intensity profiles. (**b**) Cross-section of relative fluorescence intensities show an almost perfect correlation of both intensity profiles and a very low background staining.

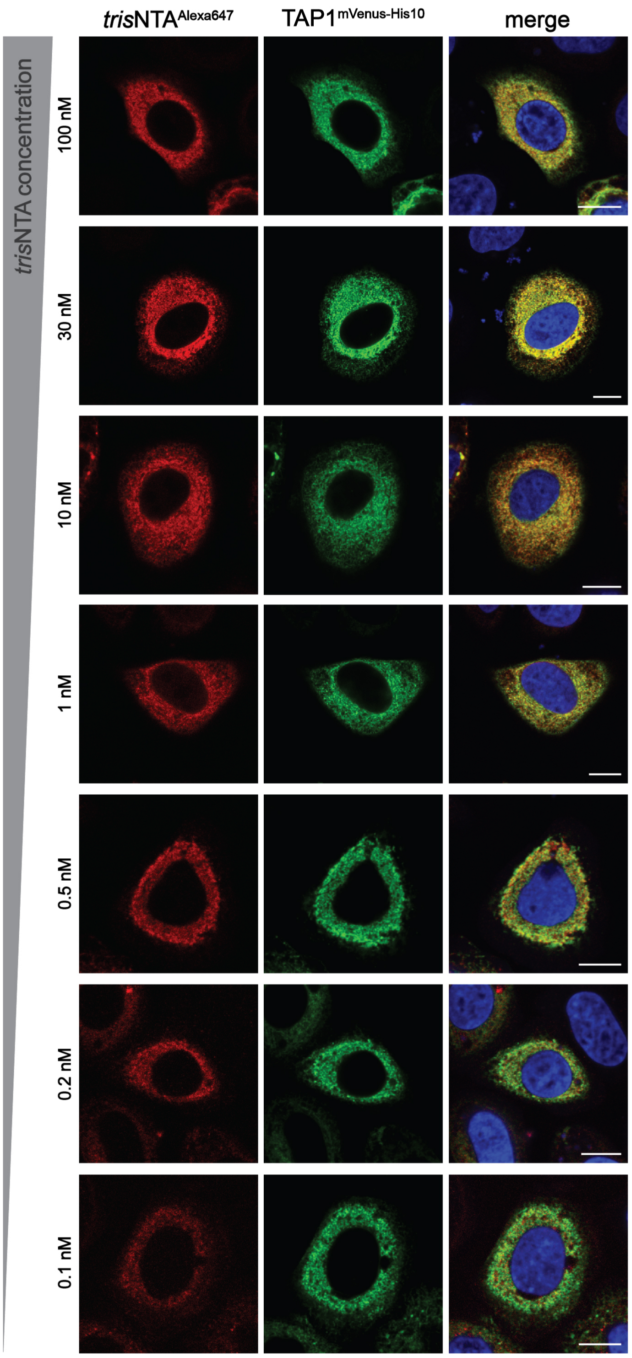

Supplementary Figure 2 ı Specific labeling of His-tagged proteins at subnanomolar concentrations. TAP1$^{mVenus-His10}$ transfected HeLa Kyoto cells (green) were stained with decreasing concentrations of *tris*NTA^{Alexa647} (100 nM - 0.1 nM, red). All applied concentrations showed specific staining of TAP1$^{mVenus-His10}$ by *tris*NTA^{Alexa647} (merge) with decreasing labeling density at subnanomolar concentrations. Even at 0.2 nM of *tris*NTA^{Alexa647} a co-localization between both reporter dyes was observed. Images were taken by CLSM. Scale bar: 10 µm.

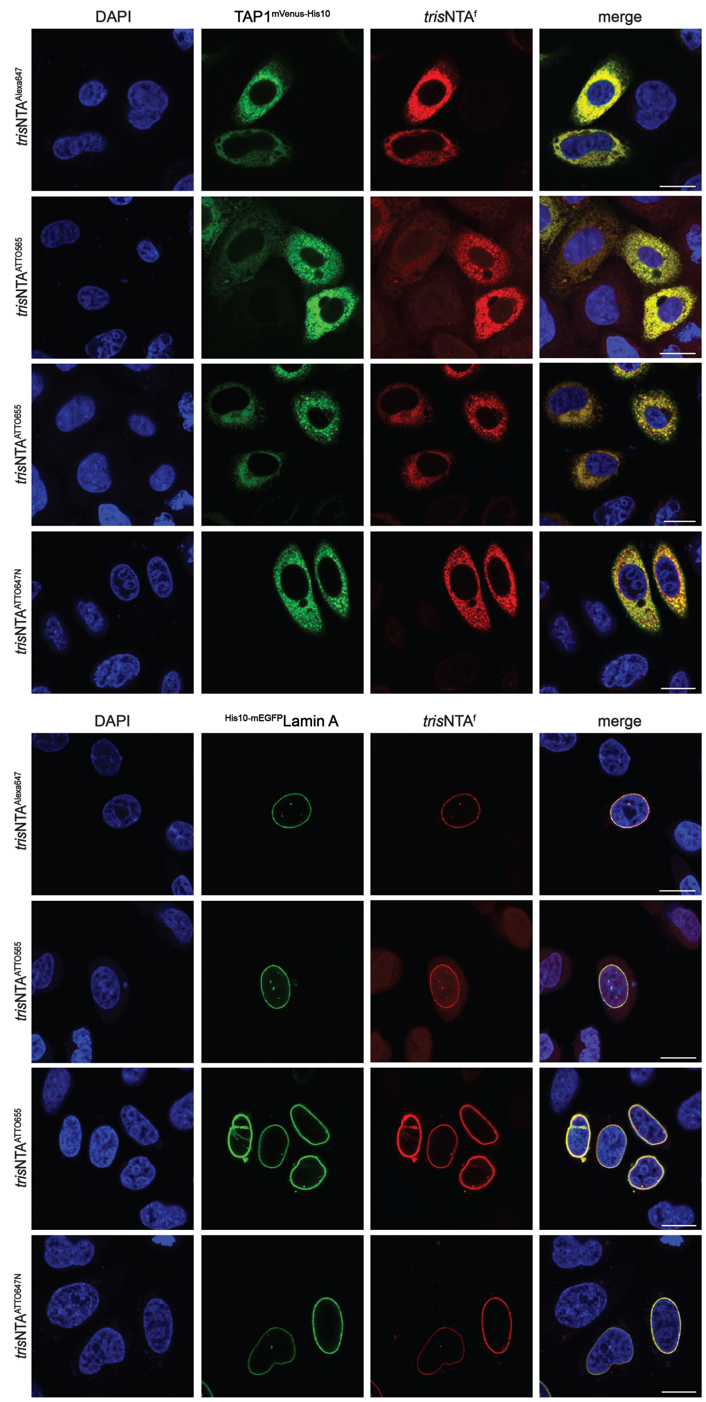

Supplementary Figure 3 ı Labeling of His-tagged proteins by different fluorescent *tris*NTAs. Cells transiently transfected with TAP1$^{mVenus\text{-}His10}$ (green) or $^{His10\text{-}mEGFP}$Lamin A (green) were chemically arrested with 4% formaldehyde and stained with *tris*NTA^{f} (100 nM), conjugated to Alexa647, ATTO565, ATTO655, or ATTO647N (from top to bottom, red). In all cases, a specific labeling of the His-tagged POI was observed, indicated by co-localization of both reporter dyes (merge). *tris*NTA^{ATTO565} targeting produced a higher background signal compared to *tris*NTA^{ATTO647N}, *tris*NTA^{Alexa647}, or *tris*NTA^{ATTO655}. Scale bar: 15 µm.

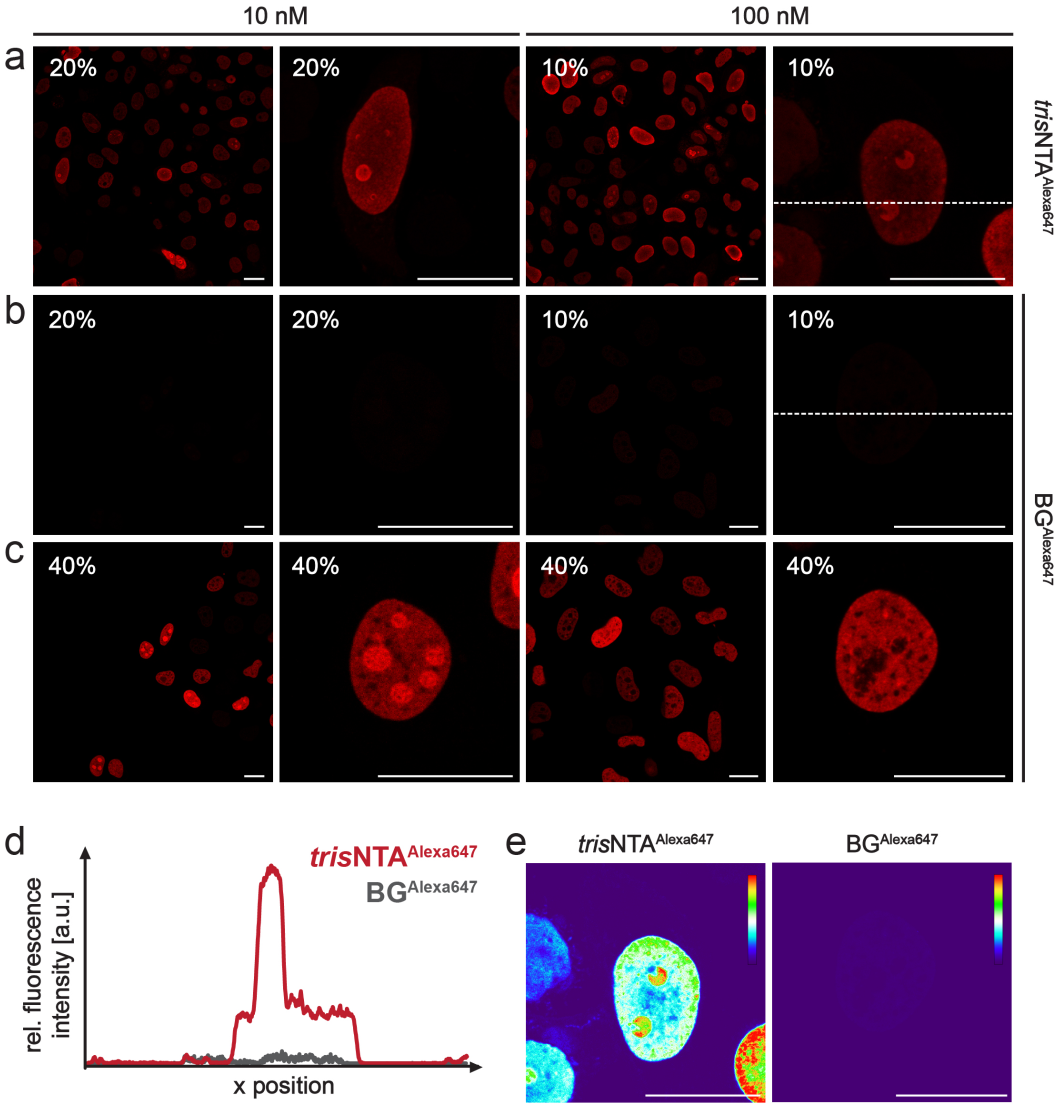

Supplementary Figure 4 ı Comparison of *tris*NTA^{f} and SNAP^{f}-tag labeling. *tris*NTA^{Alexa647} labeling of H2B$^{mVenus\text{-}His10}$ transfected cells and BG^{Alexa647} labeling of H2B^{SNAPf} transfected cells was conducted in parallel with identical concentrations and identical incubation times. (**a**, **b**) Imaging was conducted by CLSM with identical settings for each concentration, respectively (laser intensity indicated in left upper corner of every image). A significant higher labeling density was observed with *tris*NTA^{Alexa647} compared to SNAP^{f}-tag labeling. SNAP^{f}-tag labeling can be visualized with increased laser intensity (**c**, same cells imaged as in b). (**d**) Normalized fluorescence intensity cells, stained with *tris*NTA^{Alexa647} and BG^{Alexa647} (both 100 nM) along horizontal dashed line (in right images of **a** and **b**). (**e**) Heat map of the fluorescence intensity. Cells were labeled with of *tris*NTA^{Alexa647} and BG^{Alexa647} (100 nM each, right images of **a** and **b**). Scale bars: 15 µm.

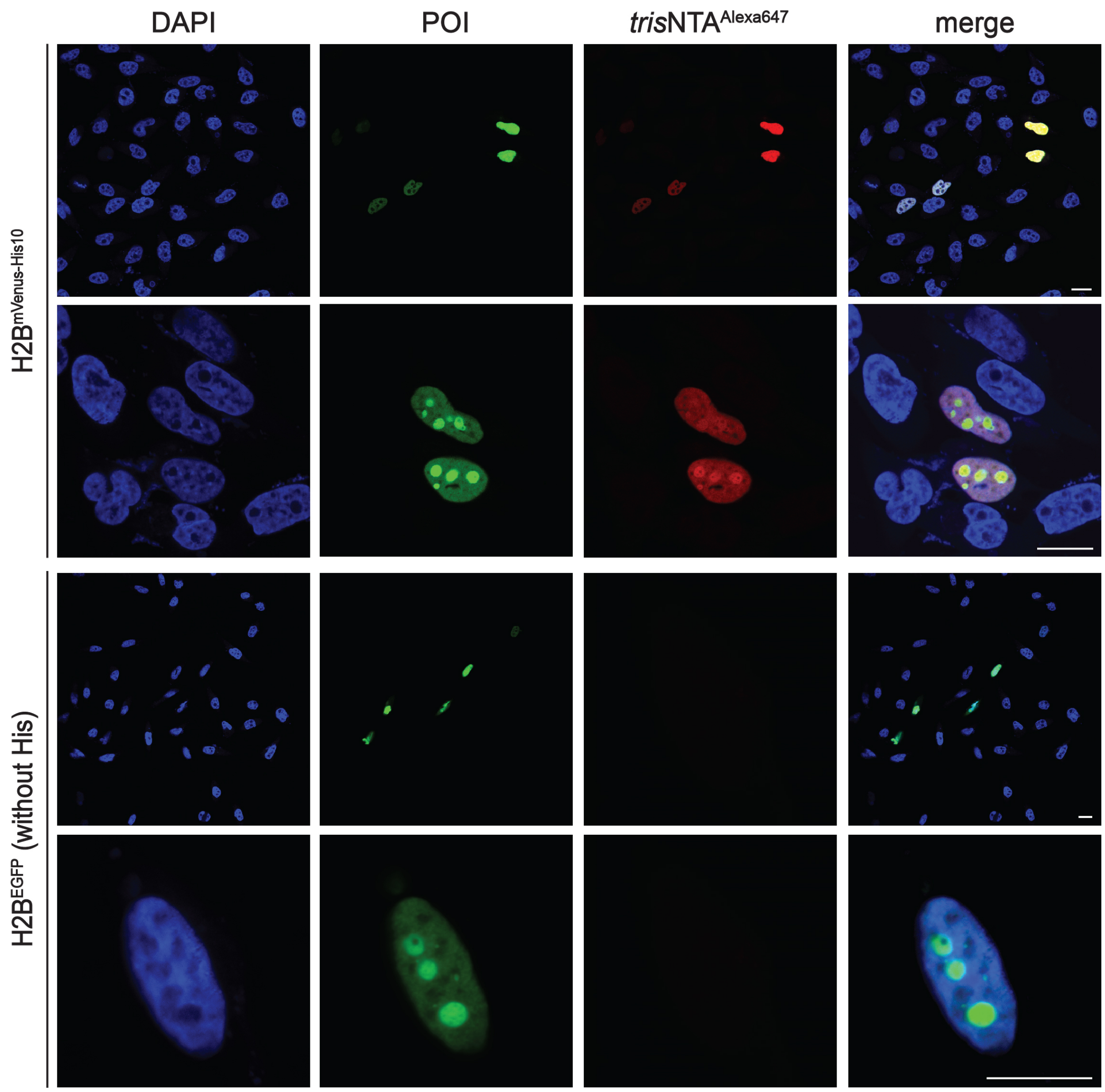

Supplementary Figure 5 ı *tris*NTA^{Alexa647} labeling is His-tag specific. After fixation, only in H2B$^{mVenus\text{-}His10}$ transfected HeLa cells co-localization of *tris*NTA^{Alexa647} (red, upper panel) and the His-tagged POI (green) was detected, whereas H2B^{EGFP} transfected cells as well as untransfected cells showed no unspecific labeling, background staining, or any signal corresponding to the fluorescence of *tris*NTA^{Alexa647} (red, lower panel). Images were recorded by CLSM. Scale bar: 10 µm.

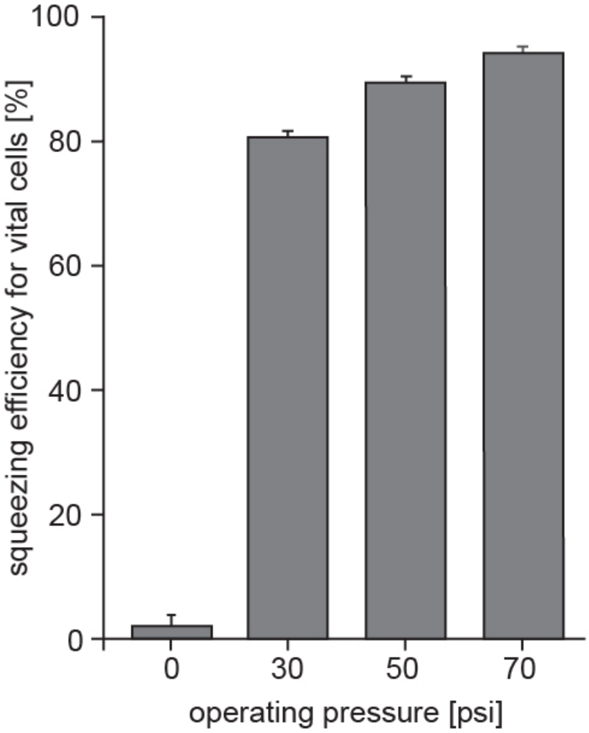

Supplementary Figure 6 ı Efficiency of cell squeezing. HeLa cells were squeezed through constrictions with 6 µm in diameter and 30 µm in length in the presence of *tris*NTA^{Alexa647} (200 nM). Analysis was performed 18 h after squeezing via flow cytometry. Dead cells were stained with propidium iodide and the efficiency of *tris*NTA^{Alexa647} uptake was exclusively analyzed for vital, unstained cells. Error bars represent the s.d. of triplicates.

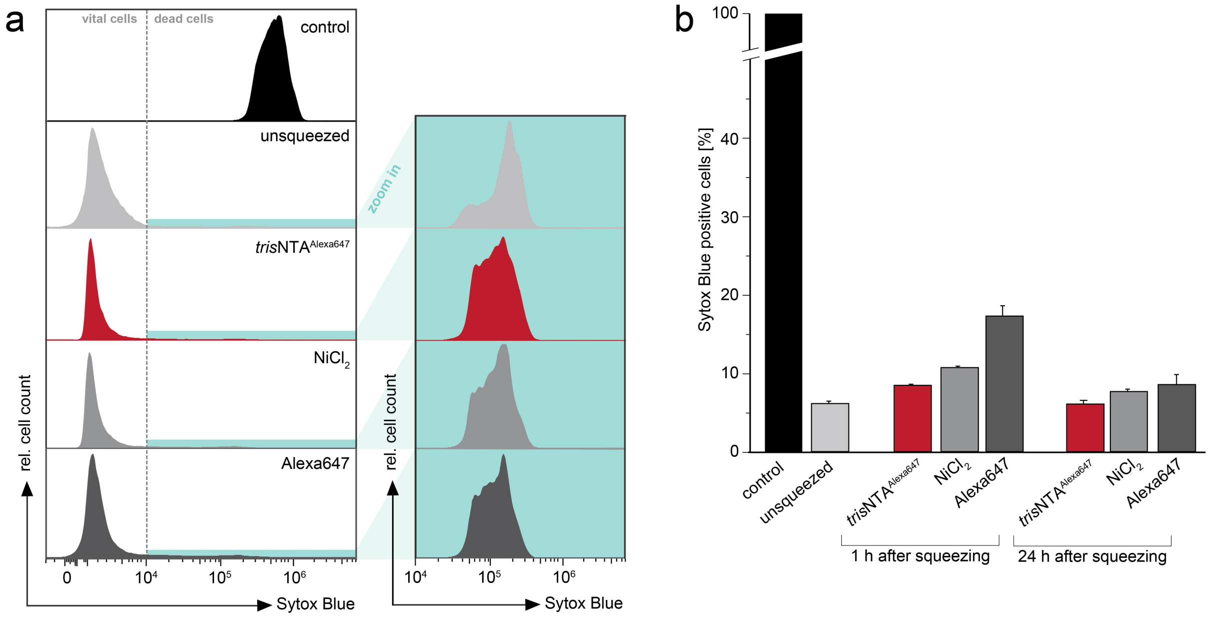

Supplementary Figure 7 | Cell viability assay of mammalian cells after squeezing. HeLa Kyoto cells were squeezed through constrictions with 7 µm in diameter and 10 µm in length in the presence of *tris*NTA^{Alexa647} (100 nM), $NiCl_2$ (500 nM) or Alexa647 (100 nM). As a control, non-squeezed cells were kept at 4 °C during the procedure. Dead cell staining was performed by 1 µM Sytox Blue 1 h (**a, b**) and 24 h (**b**) after squeezing, followed by analysis via flow cytometry (50.000 cells per sample). As a control for dead cells, HeLa Kyoto cells were permeabilized with listeriolysin O. Experiments were performed in triplicates. The mean and standard deviation is presented. (**a**) Histograms of flow cytometry data obtained 1 h after squeezing in the presence of *tris*NTA^{Alexa647}, $NiCl_2$ or Alexa647, are shown exemplarily. (**b**) Bar diagram showing the cell viability 1 h and 24 h after transduction. After 24 h, only 6.0 ± 0.5%, 7.6 ± 0.3% and 8.5 ± 1.3% of cells were stained by Sytox Blue for *tris*NTA^{Alexa647}, $NiCl_2$ as well as Alexa647 transduced cells. Intracellular delivery of *tris*NTA^{Alexa647}, $NiCl_2$ or Alexa647 by squeezing does not affect cell viability. Error bars represent the s.d. of triplicates.

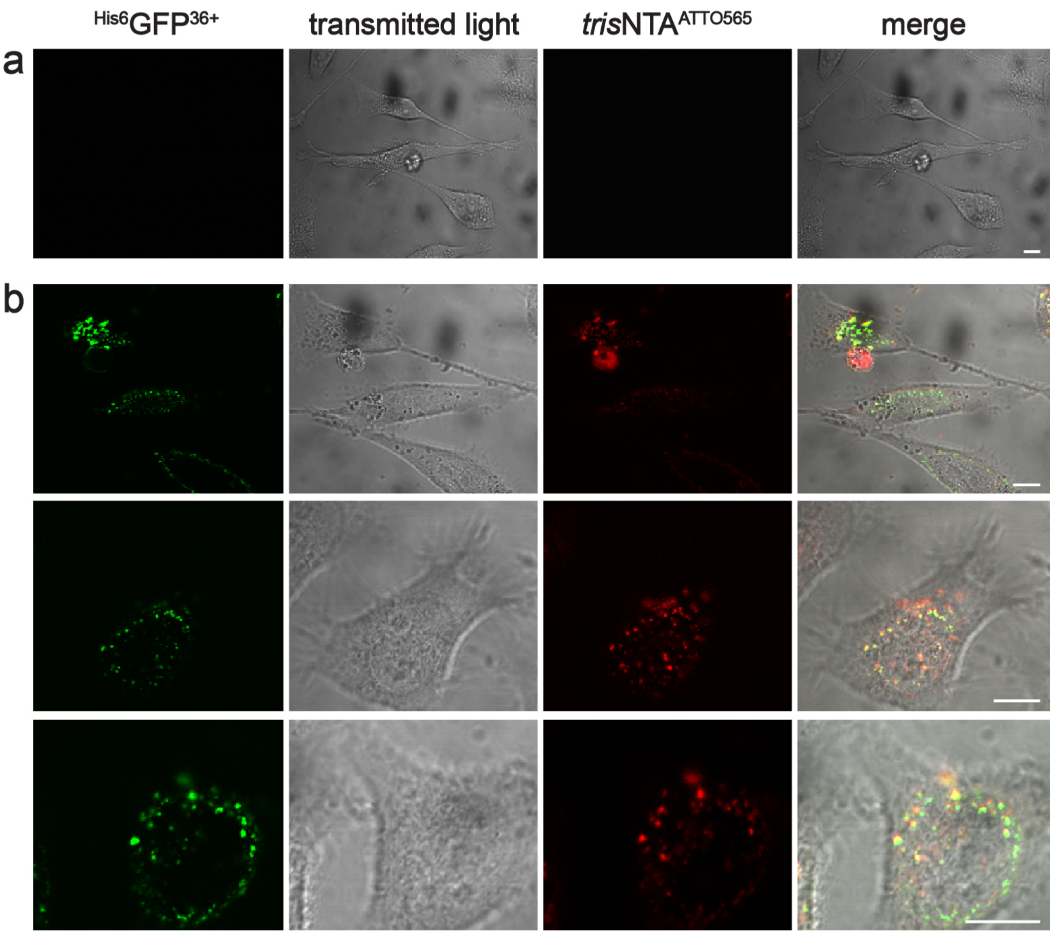

Supplementary Figure 8 ı *tris*NTA^{f} delivery via supercharged GFP. HeLa cells were incubated with a non-covalent complex of ^{His6}GFP^{36+} and *tris*NTA^{ATTO565} for intracellular delivery. (**a**) Cells before addition of the complex. **(b**) 20 min after addition of the complex, endocytic uptake of the complex, but no cytosolic distribution of *tris*NTA^{ATTO565} was observed. Scale bar: 10 µm.

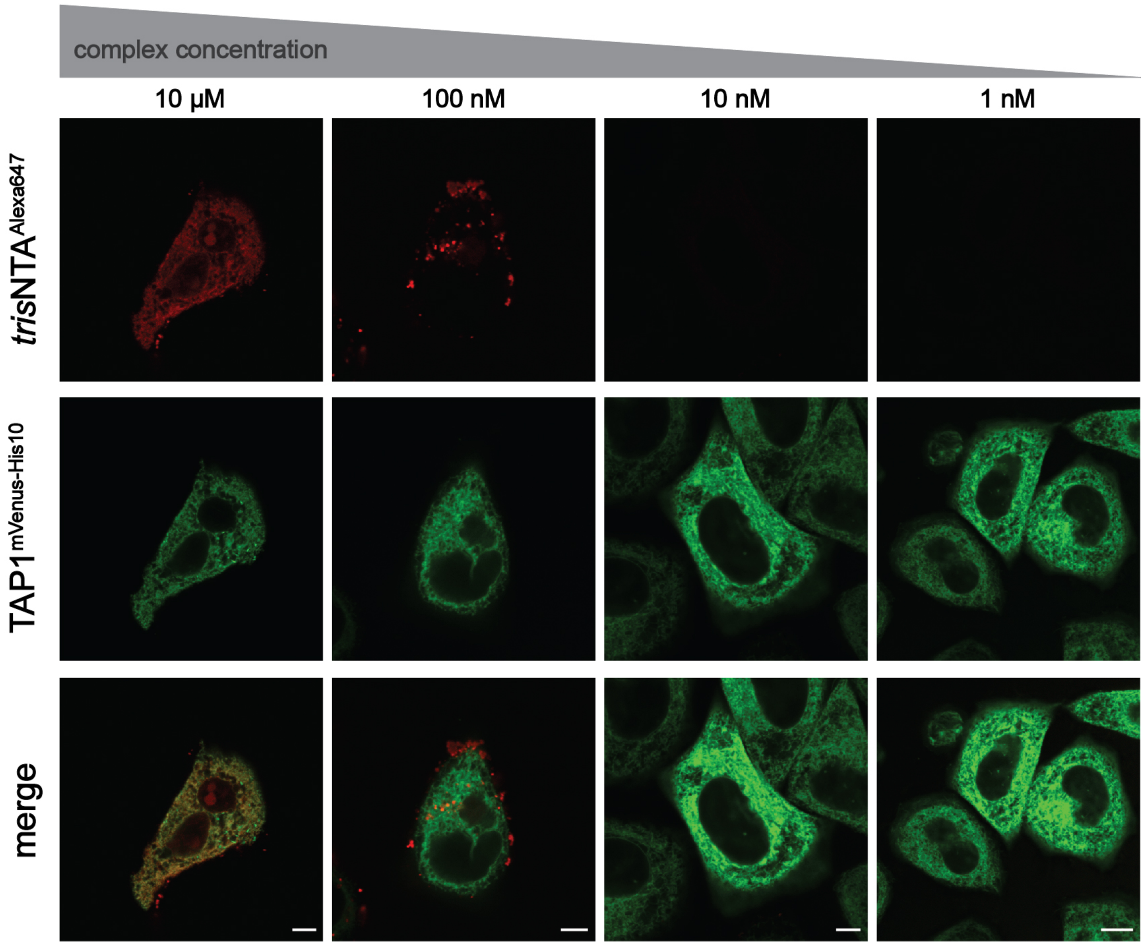

Supplementary Figure 9 ı Live-cell labeling of His-tagged proteins via delivery of *tris*NTA^{Alexa647}/Tat$_{49\text{-}57}$ carrier complex. TAP1$^{mVenus\text{-}His10}$ transfected cells were incubated with various concentrations of a non-covalent *tris*NTA^{Alexa647}/Tat$_{49\text{-}57}^{His6}$ carrier complex for intracellular delivery. Only if the carrier complex is applied at a concentration of 10 µM, a delivery and intracellular labeling is achieved, whereas with nanomolar concentrations, neither cytosolic uptake nor TAP1$^{mVenus\text{-}His10}$ labeling was accomplished. Images were recorded by CLSM. Scale bar: 5 µm.

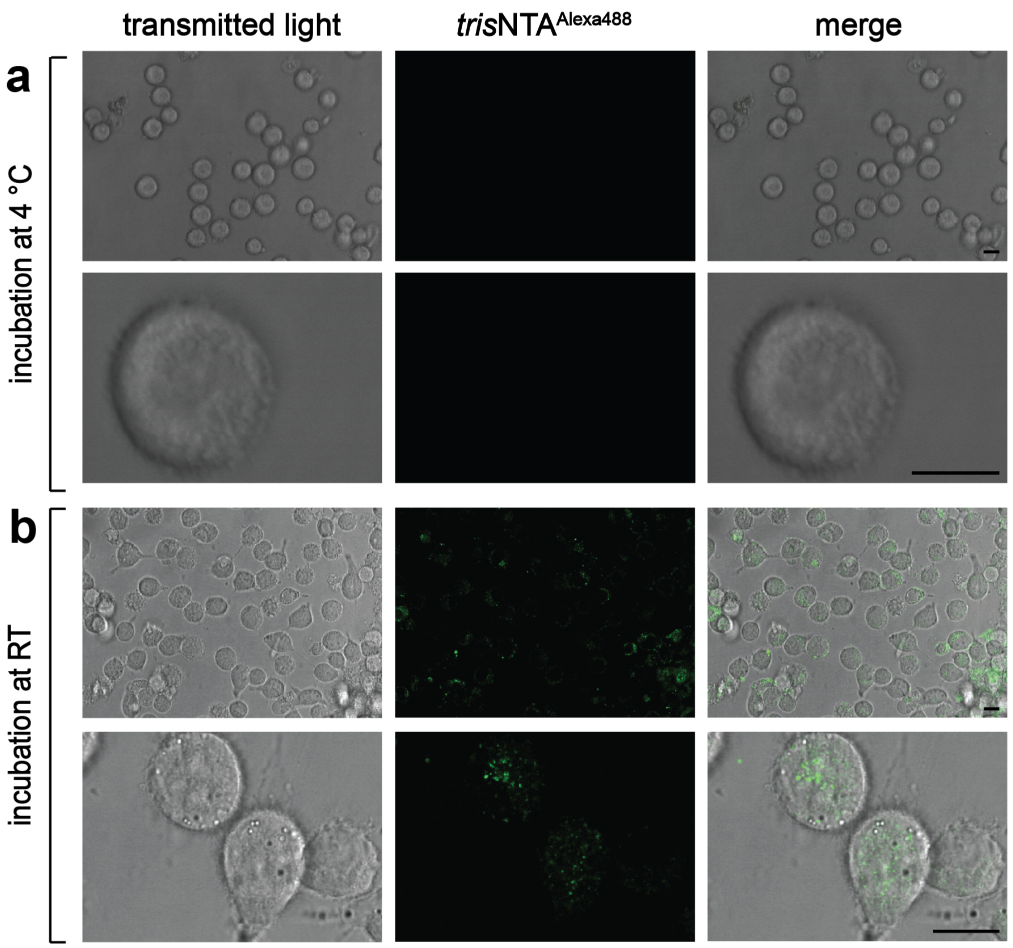

Supplementary Figure 10 ׀ Energy-dependent endocytosis was not observed at low temperature. HeLa cells were incubated with 100 nM *tris*NTA^{Alexa488} for 30 min at 4 °C (**a**) or RT (**b**). Endocytic uptake of fluorophore-labeled *tris*NTA was not detected at 4 °C (merge, **a**) compared to RT treated cells (merge, **b**), demonstrating the prevention of energy-dependent endocytic uptake of *tris*NTA^{Alexa488} at 4 °C. Images were acquired by CLSM 1 h after *tris*NTA treatment. Scale bar: 10 µm

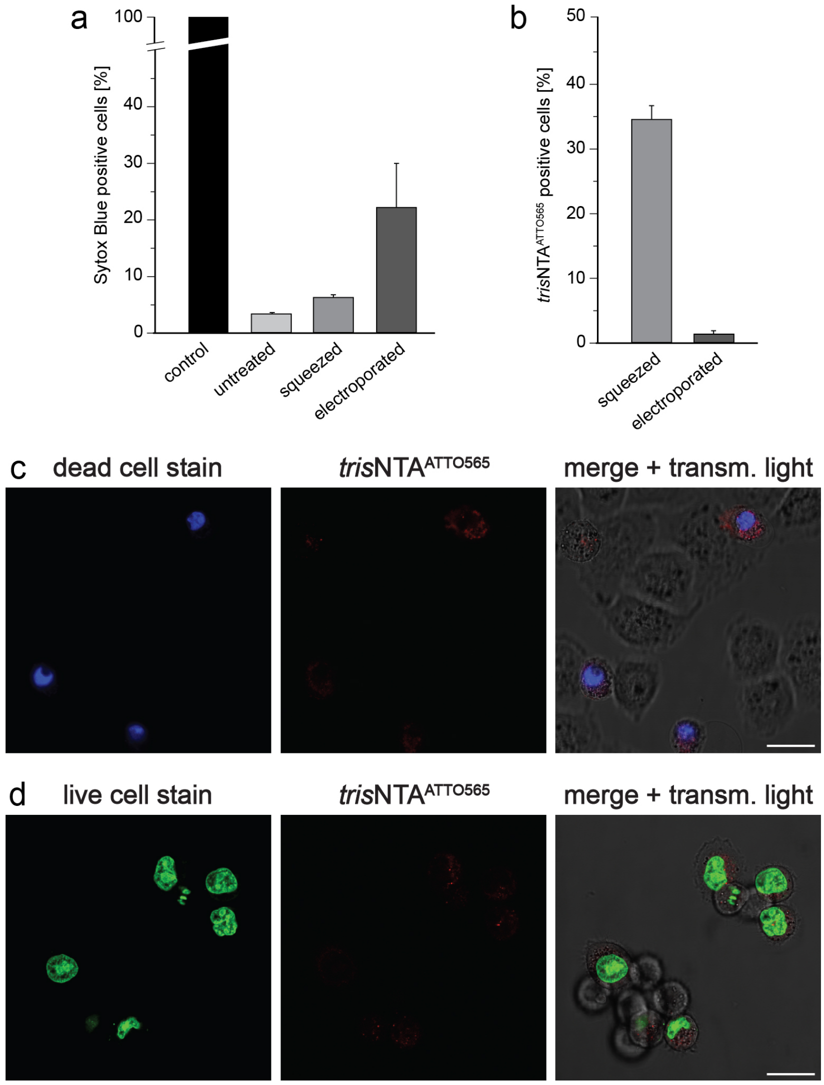

Supplementary Figure 11 ı *tris*NTA^{f} delivery by electroporation. HeLa cells were semi-permeabilized by electroporation in the presence of *tris*NTA^{ATTO565} (100 nM). (**a**) Sytox Blue Dead Cell Staining 1 h after electroporation and subsequent flow cytometry analysis revealed 21% dead cells compared to only 6% after cell squeezing. As a control, cells were semi-permeabilized with listeriolysin O. Error bars represent the s.d. of triplicates. (**b**) Cargo loading of cells was analyzed 1 h after electroporation by flow cytometry. In comparison to 35% *tris*NTA^{ATTO565} uptake by squeezing, cargo electroporation showed only a negligible uptake of 1%. Error bars indicate the s.d. of triplicates. (**c**, **d**) Dead cell staining (Sytox Blue, **c**) or live cell staining (Syto16, **d**) was performed on electroporated HeLa cells. CLSM imaging revealed low signals and a dotty distribution for *tris*NTA^{ATTO565} uptake in individual cells. Scale bar: 20 µm.

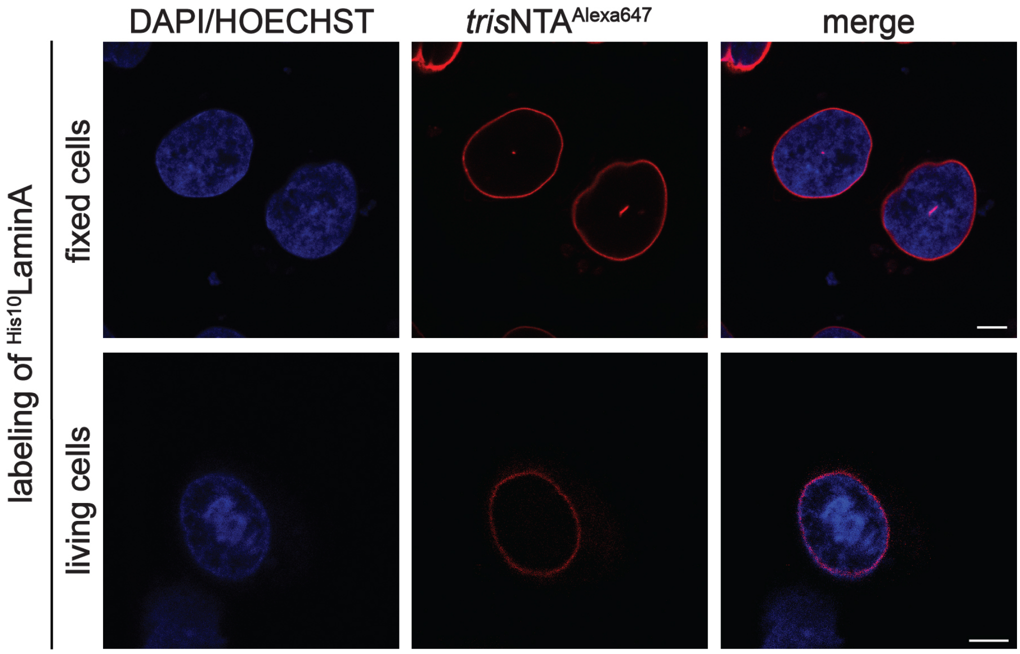

Supplementary Figure 12 ı Specific labeling of His-tagged Lamin A in fixed and living cells. Cells, transiently transfected with ^{His10}Lamin A carrying no bulky fluorescent proteins, were fixed with 4% formaldehyde and stained with 100 nM *tris*NTA^{Alexa647} (red, top) as well as DAPI (blue, top). *tris*NTA^{Alexa647} specifically stains ^{His10}Lamin A at the nuclear envelope (merge, top). For labeling in living cells, *tris*NTA^{Alexa647} (red, bottom) was squeezed into cells transfected with ^{His10}Lamin A (bottom). Imaging after HOECHST staining (blue, bottom) showed a specific labeling of His-tagged Lamin A at the nuclear envelope (merge, bottom). Images were taken by CLSM. Scale bar: 5 µm.

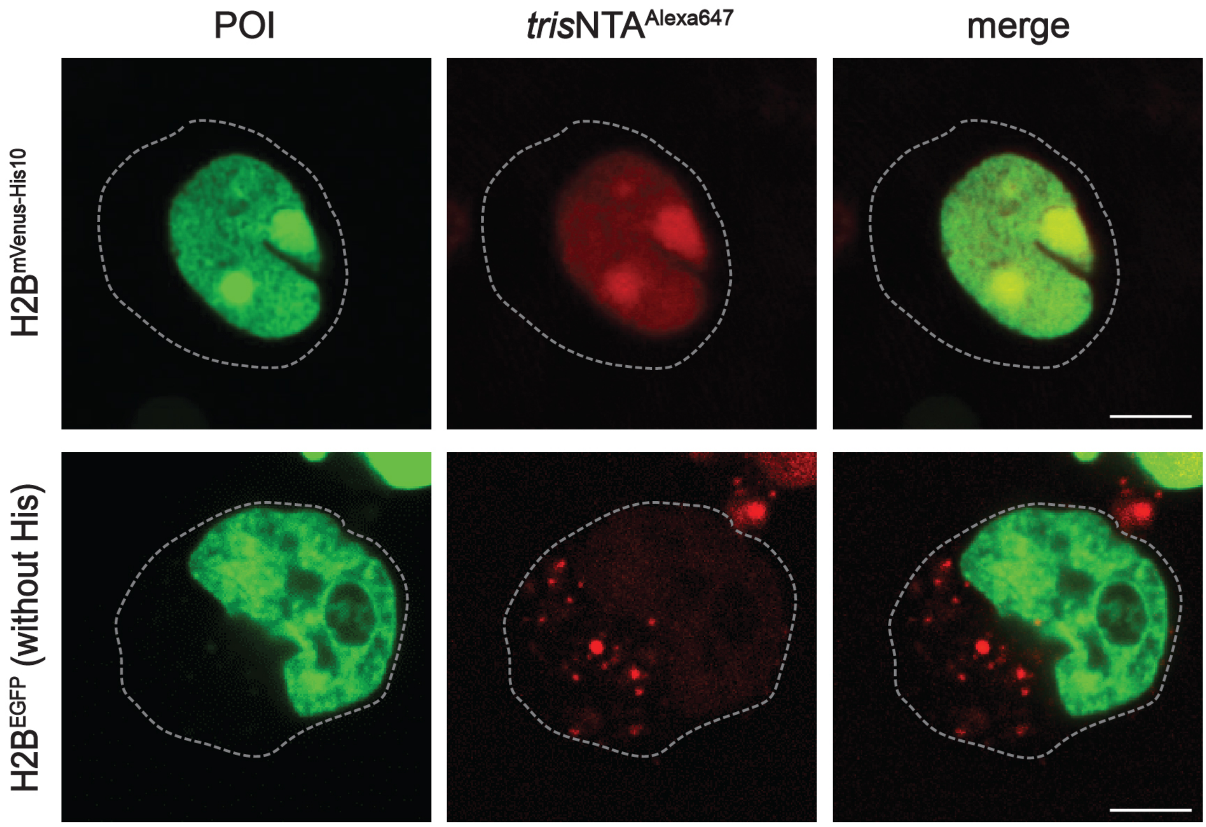

Supplementary Figure 13 ǀ Specific labeling of His-tagged H2B by *tris*NTA$^{\mathbf{Alexa647}}$ in living cells. After squeezing in the presence of *tris*NTA^{Alexa647} (100 nM), only in H2B$^{mVenus\text{-}His10}$ transfected HeLa cells, co-localization of *tris*NTA^{Alexa647} (red, upper panel) and the POI (green) was detected. In H2B^{EGFP} transfected cells, no unspecific labeling was observed for *tris*NTA^{Alexa647} (red, lower panel), apart from negligible background staining. This confirms the high specificity of *tris*NTA^{Alexa647} for His-tagged proteins in living cells. Dashed lines indicate the cell border. Scale bar: 10 µm.

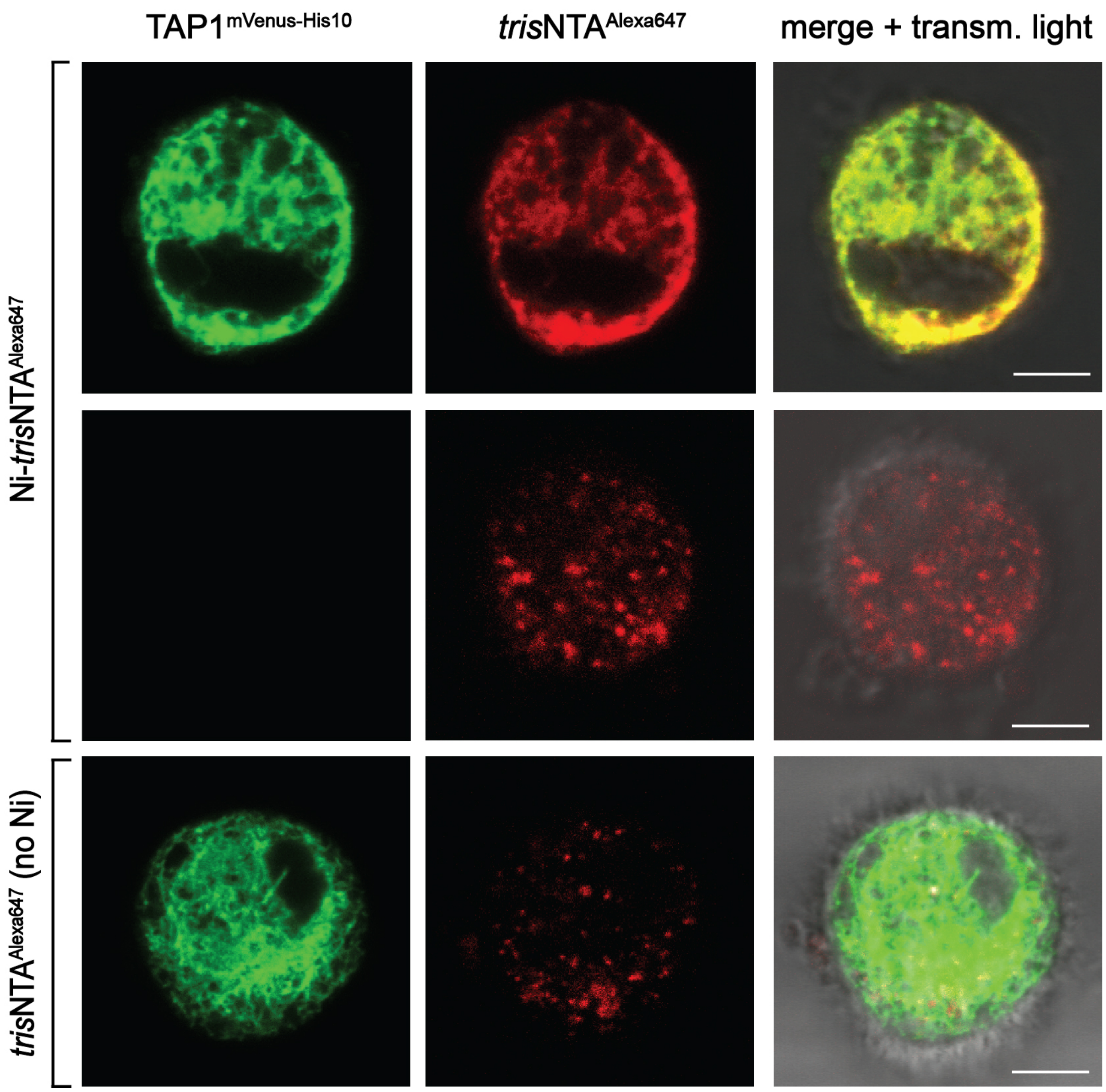

Supplementary Figure 14 ׀ Specific labeling of His-tagged TAP1 with *tris*NTA^{Alexa647} in living cells. *tris*NTA^{Alexa647} was delivered to TAP1$^{mVenus-His10}$ transfected HeLa cells by squeezing. Only *tris*NTA^{Alexa647} in complex with Ni(II) showed co-localization with TAP1$^{mVenus-His10}$ (merge, upper panel) at the endoplasmic reticulum (ER) membrane. A punctual distribution of Ni-*tris*NTA^{Alexa647} (red, middle panel) was observed in the absence of His-tagged TAP1 (green, middle panel) without unspecific labeling of the ER. No co-localization between His-tagged TAP1 (green, lower panel) and *tris*NTA^{Alexa647} (red, lower panel) lacking Ni(II) was observed, indicating the specific interaction as well as necessity of Ni(II) for the *lock-and-key* probe. Images were recorded 1 h after squeezing by CLSM. Scale bar: 5 µm.

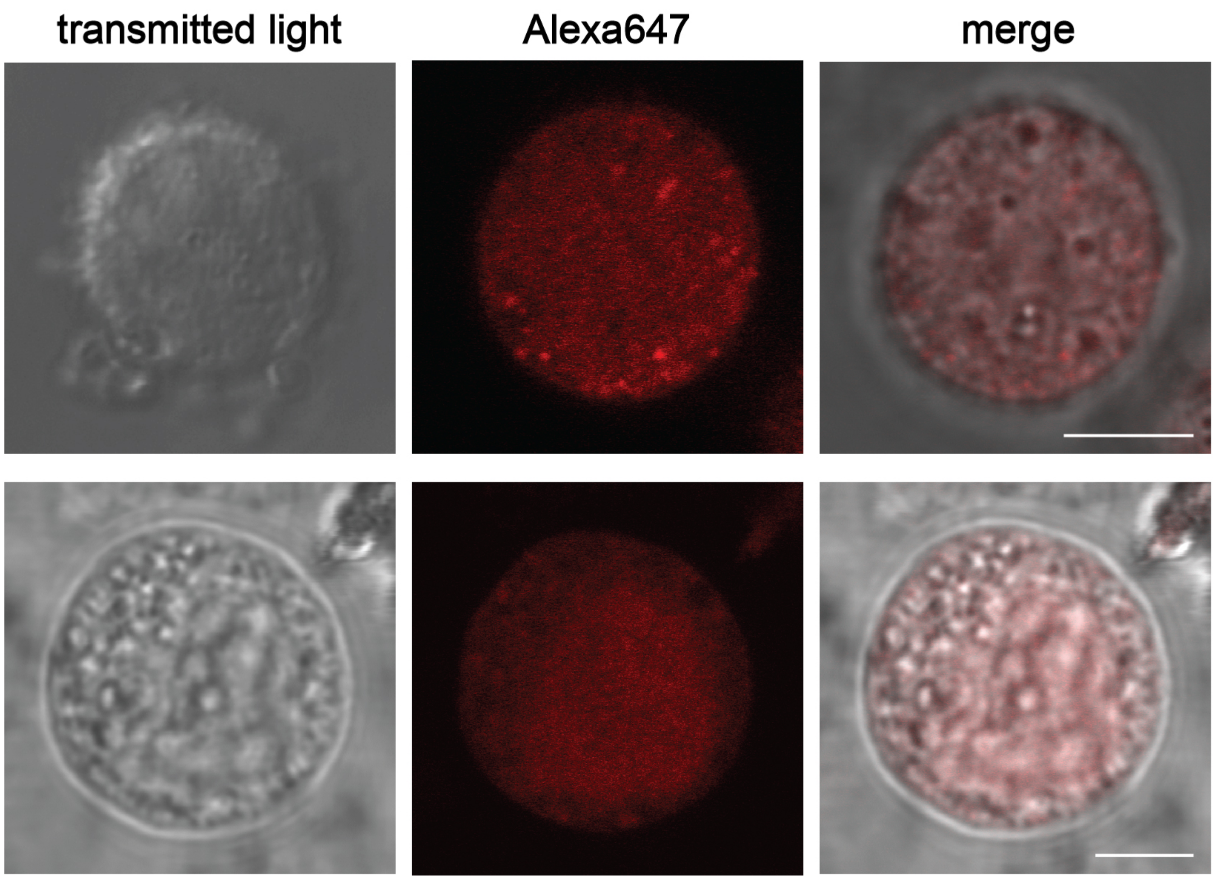

Supplementary Figure 15 ı Cytosolic distribution of Alexa647 in living cells. 100 nM of Alexa647 was delivered to HeLa Kyoto cells by squeezing. Apart from punctuated structures, a cytosolic distribution of the dye was detected (red) in all squeezing experiments. No distinct subcellular localization or unspecific staining of Alexa647 itself was observed 1 h after squeezing, supporting the specific interaction of the *lock-and-key* probe. Imaging was performed by CLSM. Scale bar: 5 μm.

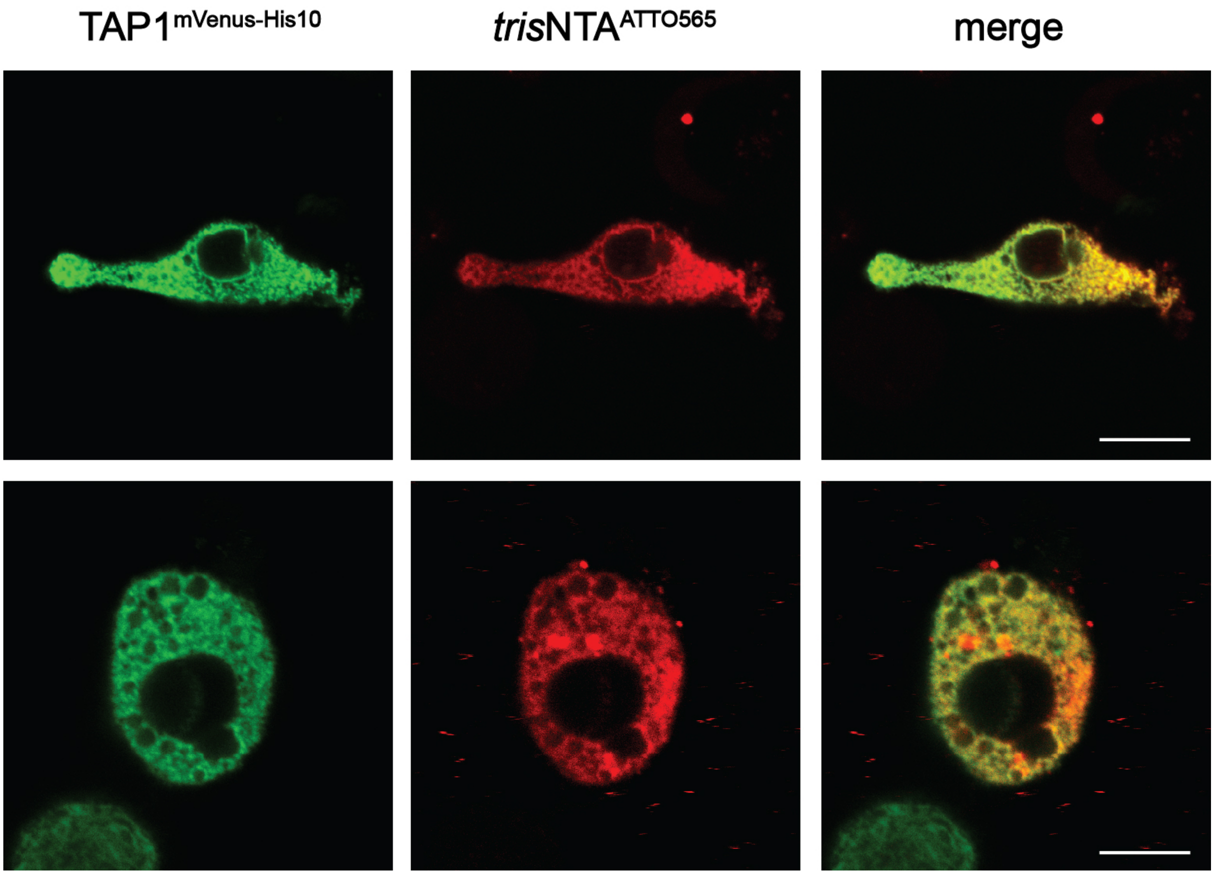

Supplementary Figure 16 ı Live-cell labeling of TAP1$^{mVenus\text{-}His10}$ by *tris*NTA^{ATTO565}. TAP1$^{mVenus\text{-}His10}$ transfected HeLa cells were squeezed in the presence of *tris*NTA^{ATTO565} (100 nM). Co-localization between *tris*NTA^{ATTO565} (red) and His_{10}-tagged TAP1 (green) was observed, demonstrating specific targeting of the His-tagged POI by *tris*NTA^{ATTO565} in vivo. This verifies the high-specificity of the *tris*NTA-His-tag interaction for in-cell labeling and is comparable to TAP1$^{mVenus\text{-}His10}$ targeting by *tris*NTA^{Alexa647} (see Fig. 2 and Supplementary Fig. 14). Images were recorded by CLSM 1 h after squeezing. Scale bar: 5 µm.

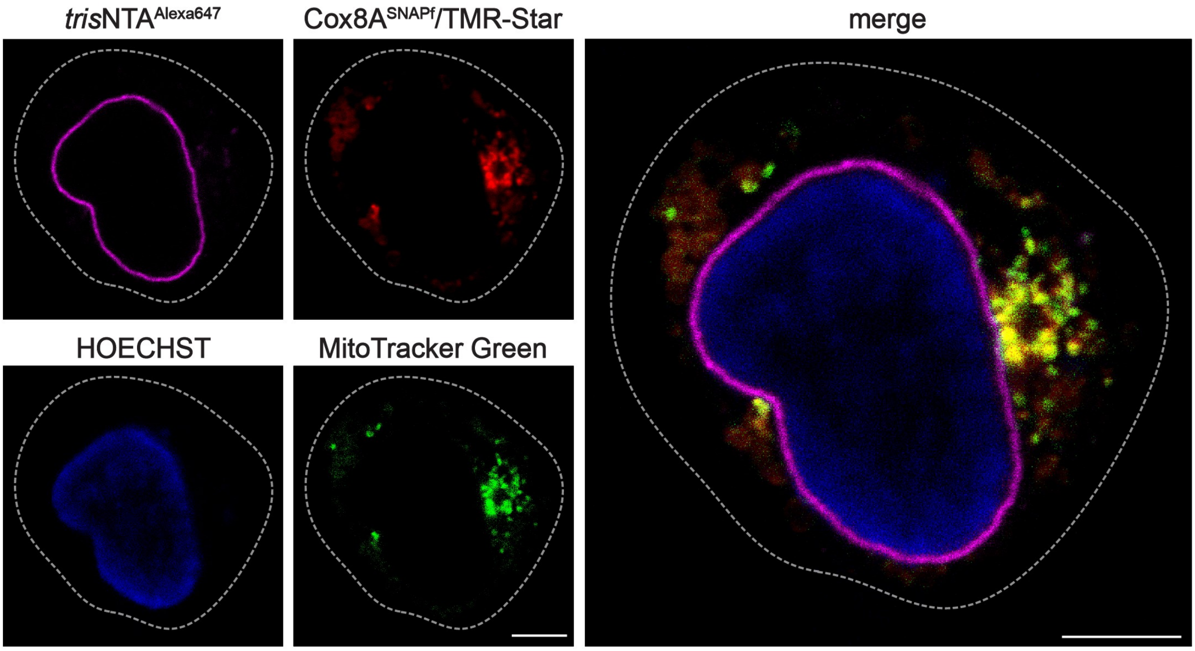

Supplementary Figure 17 ı Combination of *tris*NTA^{f} labeling with SNAP^{f}-tag labeling in living cells. HeLa Kyoto cells, co-expressing ^{His10}LaminA and the SNAP^{f}-tagged cytochrome c oxidase subunit 8A (Cox8A-SNAP^{f}) were squeezed with 100 nM *tris*NTA^{Alexa647} and subsequently incubated with cell permeable TMR-Star for SNAP^{f}-tag labeling. Confocal images were taken 1 h after squeezing. Specific labeling of ^{His10}LaminA by *tris*NTA^{Alexa647} (magenta) and simultaneous labeling of Cox8A-SNAP^{f} by TMR-Star (red) was detected, whereas Cox8A labeling co-localizes with a mitochondrial marker (green). Dashed lines indicate the cell border. Scale bar: 5 µm.
